# Astilbin Alleviates IL-17-Induced Hyperproliferation and Inflammation in HaCaT Cells via Inhibiting Ferroptosis Through the cGAS-STING Pathway

**DOI:** 10.3390/ijms26115075

**Published:** 2025-05-24

**Authors:** Xiaohan Xu, Huizhong Zhang, Aqian Chang, Hulinyue Peng, Shiman Li, Ke Zhang, Wenqi Wang, Xingbin Yin, Changhai Qu, Xiaoxv Dong, Jian Ni

**Affiliations:** School of Chinese Material Medica, Beijing University of Chinese Medicine, Beijing 102488, China; 20230935144@bucm.edu.cn (X.X.); 20230935145@bucm.edu.cn (H.Z.); 20220941483@bucm.edu.cn (A.C.); 20230941504@bucm.edu.cn (H.P.); 20230935146@bucm.edu.cn (S.L.); 20240935273@bucm.edu.cn (K.Z.); 20240935097@bucm.edu.cn (W.W.); yxbtcm@bucm.edu.cn (X.Y.); quchanghai@bucm.edu.cn (C.Q.)

**Keywords:** Astilbin, psoriasis, ferroptosis, cGAS-STING pathway, proinflammatory cytokine

## Abstract

Psoriasis, a chronic inflammatory skin disorder, is driven by dysregulated immune responses and keratinocyte dysfunction. Here, we explore the therapeutic potential of Astilbin (AST), a flavonoid with potent anti-inflammatory properties, in modulating ferroptosis and the cyclic GMP-AMP synthase (cGAS)-stimulator of interferon genes (STING) pathway in IL-17-stimulated HaCaT keratinocytes. Our psoriatic cell model recapitulated key pathological features, including hyperproliferation, membrane integrity loss, mitochondrial dysfunction, and heightened oxidative stress, alongside elevated proinflammatory cytokine levels. Ferroptosis-related biomarkers were significantly altered, with increased malondialdehyde (MDA) accumulation, reduced glutathione (GSH) levels, iron overload (Fe^2+^), and enhanced lipid peroxidation (detected via C11-BODIPY). Mechanistically, mitochondrial damage triggered cytoplasmic leakage of mitochondrial DNA (mtDNA), activating the cGAS-STING pathway, as evidenced by upregulated pathway-associated protein expression. AST intervention effectively mitigated these pathological changes by suppressing ferroptosis and modulating cGAS-STING signaling. These findings reveal a dual-pathway regulatory mechanism, positioning AST as a promising therapeutic candidate for psoriasis. By elucidating the interplay between ferroptosis and the cGAS-STING pathway, this study provides new insights into psoriatic inflammation and offers a rationale for targeting these pathways in therapeutic strategies.

## 1. Introduction

Psoriasis, a chronic immune-mediated skin disease, is characterized by epidermal hyperproliferation, aberrant keratinocyte differentiation, and dermoepidermal infiltration of immune cells, including monocytes, dendritic cells, and T lymphocytes [1,2,3]. These processes lead to vascular dilation and pathological tissue remodeling [4,5]. The pathogenesis of psoriasis involves genetic predisposition, immune dysregulation—particularly T helper 17 (Th17) cell polarization and interleukin-23/interleukin-17 (IL-23/IL-17) axis activation—cytokine network imbalances, keratinocyte dysfunction, and cascades of inflammatory mediators [6,7,8,9,10,11,12,13].

Emerging evidence highlights the role of ferroptosis, an iron-dependent form of regulated cell death marked by iron overload, lipid peroxidation, and antioxidant system failure [14,15,16,17]. Ferroptosis contributes to disease progression through membrane destabilization and the release of pro-inflammatory factors [18,19,20]. Conversely, inflammatory microenvironments can exacerbate ferroptosis via reactive oxygen species (ROS)-mediated lipid peroxidation, creating a pathogenic feedback loop [21].

The cGAS-STING signaling axis, a critical component of innate immunity, is activated by cytosolic DNA [22]. Upon recognizing mitochondrial DNA (mtDNA) released during cellular stress, cGAS synthesizes cyclic GMP-AMP (cGAMP), triggering STING activation, TANK-binding kinase 1 (TBK1) phosphorylation, and interferon regulatory factor 3 (IRF3)-mediated type I interferon responses [23,24]. Hyperactivation of this pathway is implicated in autoimmune diseases, including psoriasis [9].

In psoriatic lesions, elevated ROS levels drive ferroptosis, characterized by lipid peroxide accumulation. This process initiates a self-amplifying cycle: lipid peroxidation exacerbates oxidative stress, disrupts iron homeostasis, and induces Fe^2+^ overload. The iron-dependent Fenton reaction further intensifies mitochondrial damage, leading to mtDNA release into the cytosol. Cytosolic mtDNA activates the cGAS-STING pathway, amplifying inflammatory cytokine and chemokine production. This interplay between ferroptosis and cGAS-STING signaling creates a pathogenic feedforward loop, where sustained inflammation promotes oxidative stress and iron dysregulation, while ferroptotic cell death releases damage-associated molecular patterns (DAMPs), further driving immune hyperactivation [25,26].

**Figure 1:**
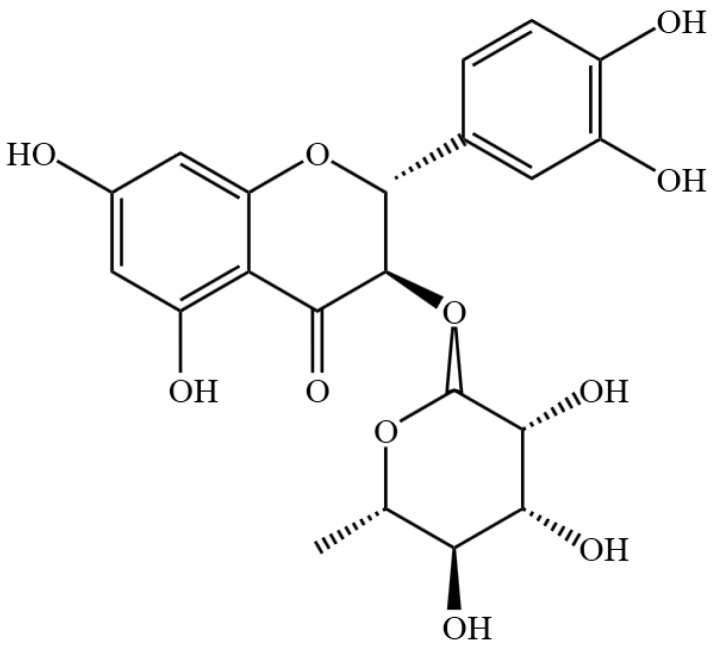
Chemical structure formula of AST.

Astilbin (AST), a bioactive flavonoid derived primarily from *Saxifragaceae* (Astilbe spp.) and *Liliaceae* species, exhibits a broad spectrum of pharmacological activities, including anti-inflammatory, antitumor, cardioprotective, antidiabetic, hepatoprotective, and antiviral effects (its structural formula is shown in Figure 1) [27,28,29,30,31,32,33]. Its anti-inflammatory properties are mediated through multiple mechanisms, such as the suppression of inflammatory mediators, modulation of signaling pathways, free radical scavenging, and immunoregulation, making AST a promising candidate for treating inflammatory disorders [27,34,35]. AST has been demonstrated to modulate the Th1/Th2 cell equilibrium, thereby attenuating the expression of key inflammatory cytokines, including tumor necrosis factor-α (TNF-α), IL-23, and IL-17A. This regulatory effect not only suppresses the hyperproliferation of keratinocytes but also ameliorates localized psoriatic lesions [36,37].

Above all, we propose that AST could attenuate IL-17-induced cGAS-STING pathway activation in HaCaT keratinocytes, consequently inhibiting cellular ferroptosis and ameliorating psoriatic pathology.

## 2. Results

### 2.1. AST Attenuated IL-17-Induced Keratinocyte Damage

**Figure 2:**
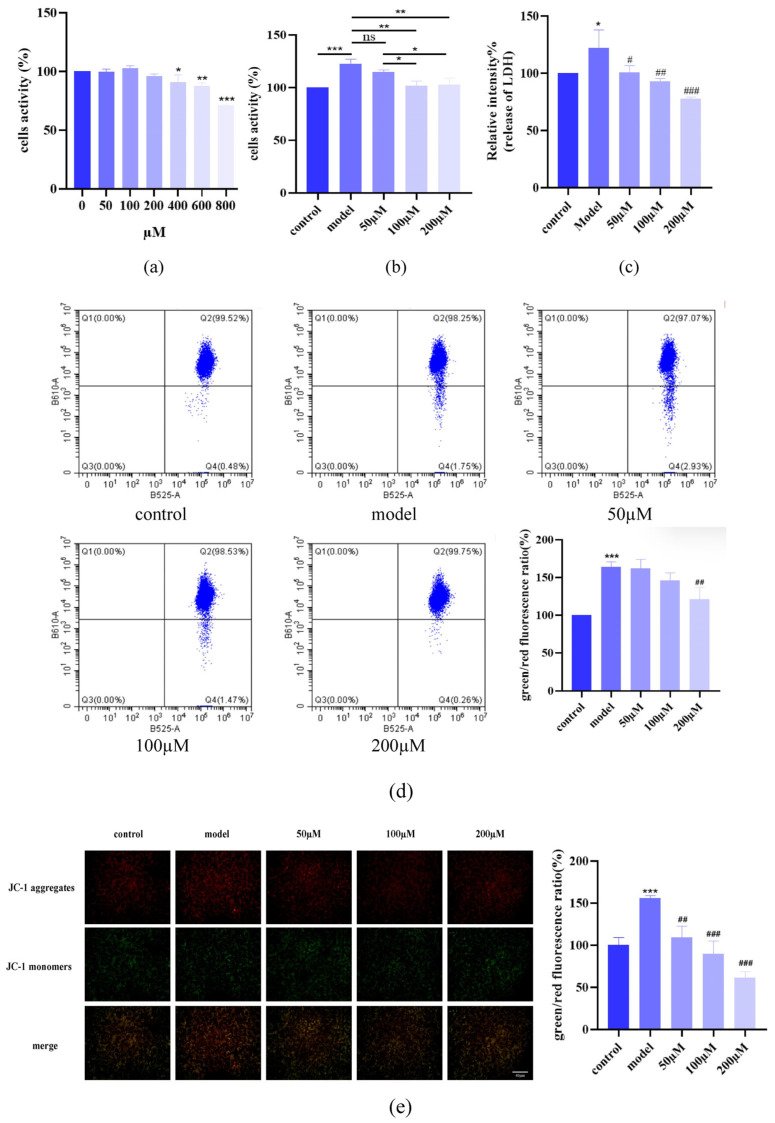
Protective effects of AST against IL-17-induced cellular damage in HaCaT cells. (**a**) Cytotoxicity assessment of AST (0–200 μM) in HaCaT cells (* *p* < 0.05, ** *p* < 0.01, *** *p* < 0.001 versus control group). (**b**) Suppression of IL-17-induced hyperproliferation by AST (* *p* < 0.05, ** *p* < 0.01, *** *p* < 0.001, “ns” denotes non-significant). (**c**) Attenuation of membrane integrity damage as measured by LDH release (* *p* < 0.05 versus control group; ^#^ *p* < 0.05, ^##^ *p* < 0.01, ^###^ *p* < 0.001 versus IL-17-induced model group). (**d**) Flow cytometric analysis of mitochondrial membrane potential using JC-1 staining, demonstrating dose-dependent restoration by AST (*** *p* < 0.001 versus control group; ^##^ *p* < 0.01 versus IL-17-induced model group). (**e**) Fluorescence microscopic visualization of JC-1 staining, showing dose-dependent reduction in green/red fluorescence intensity ratio (*** *p* < 0.001 versus control group; ^##^ *p* < 0.01, ^###^ *p* < 0.001 versus IL-17-induced model group). Data are expressed as mean ± SD (*n* = 3).

To assess AST’s anti-inflammatory effects, HaCaT cells were stimulated with IL-17 (120 ng/mL, 24 h) to establish a psoriatic inflammation model. AST treatment (0–200 μM, 24 h) exhibited concentration-dependent cytoprotection, with no cytotoxicity observed at ≤200 μM (Figure 2a). Methylthiazolyldiphenyl-tetrazolium bromide (MTT) assays demonstrated that AST suppressed IL-17-induced hyperproliferation (Figure 2b), reduced LDH release (indicative of membrane integrity restoration; Figure 2c), and restored mitochondrial membrane potential (JC-1 assay; Figure 2d,e). These results indicated that AST mitigated IL-17-mediated cellular damage, likely through mitochondrial stabilization and membrane protection.

### 2.2. AST Reduced Oxidative Stress in IL-17-Stimulated Keratinocytes

**Figure 3:**
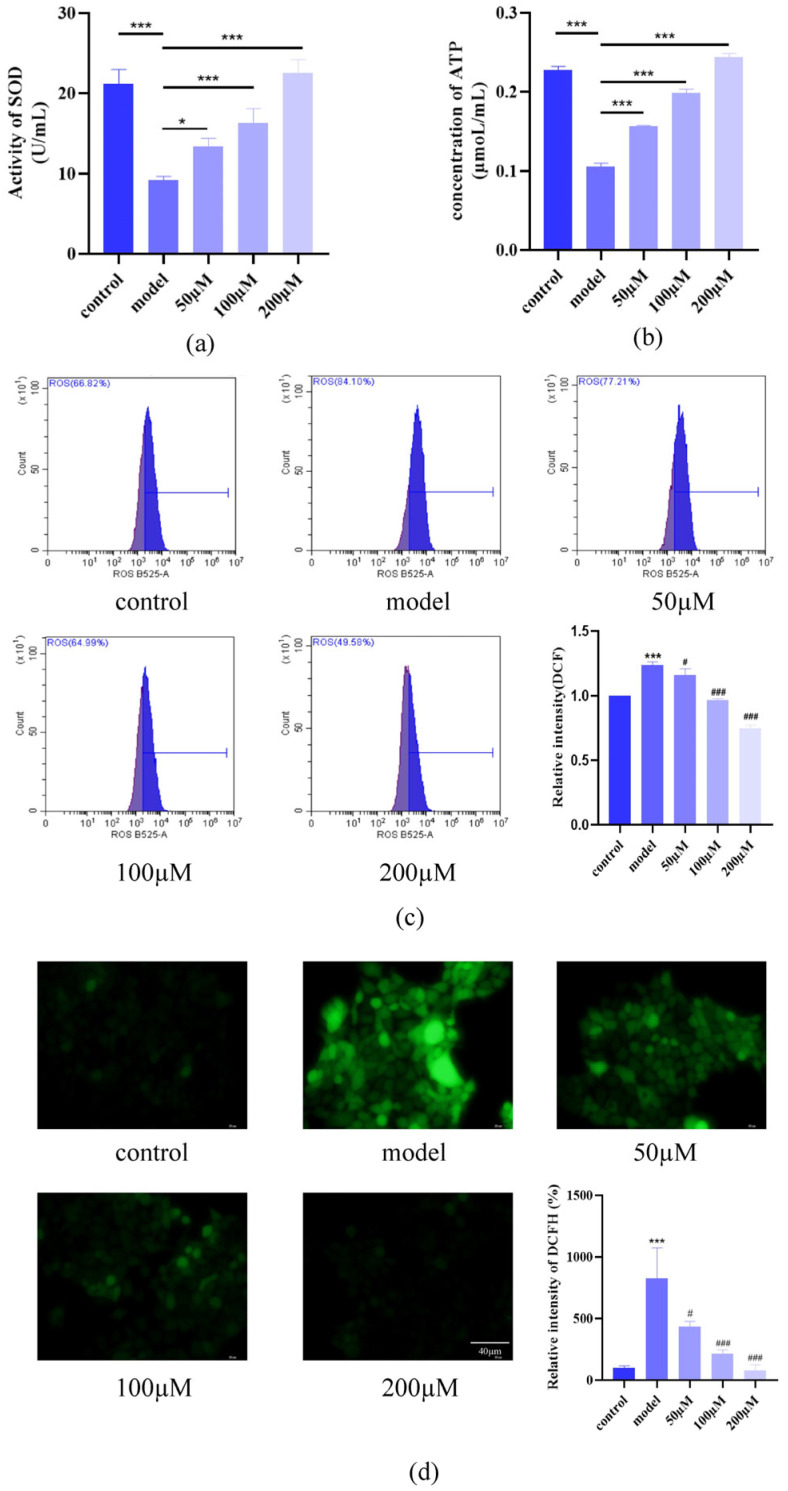
AST attenuates IL-17-induced oxidative stress in HaCaT cells. (**a**) Dose-dependent restoration of SOD activity by AST in IL-17-stimulated cells (* *p* < 0.05, *** *p* < 0.001). (**b**) Dose-dependent enhancement of intracellular ATP levels by AST treatment (*** *p* < 0.001). (**c**) Flow cytometric quantification of ROS using DCFH-DA staining (*** *p* < 0.001 versus control group; ^#^ *p* < 0.05, ^###^ *p* < 0.001 versus IL-17-induced model group). (**d**) Fluorescence microscopic visualization of ROS levels following DCFH-DA staining (*** *p* < 0.001 versus control group; ^#^ *p* < 0.05, ^###^ *p* < 0.001 versus IL-17-induced model group). Data are expressed as mean ± SD (*n* = 3).

IL-17 exposure significantly decreased Superoxide dismutase (SOD) activity and Adenosine triphosphate (ATP) levels while elevating ROS accumulation, reflecting oxidative stress and mitochondrial dysfunction. AST treatment restored SOD activity (Figure 3a), normalized ATP content (Figure 3b), and reduced ROS levels in a dose-dependent manner (flow cytometry: Figure 3c; fluorescence microscopy: Figure 3d), highlighting its antioxidative capacity.

### 2.3. AST Modulated Inflammatory Cytokine Secretion

**Figure 4:**
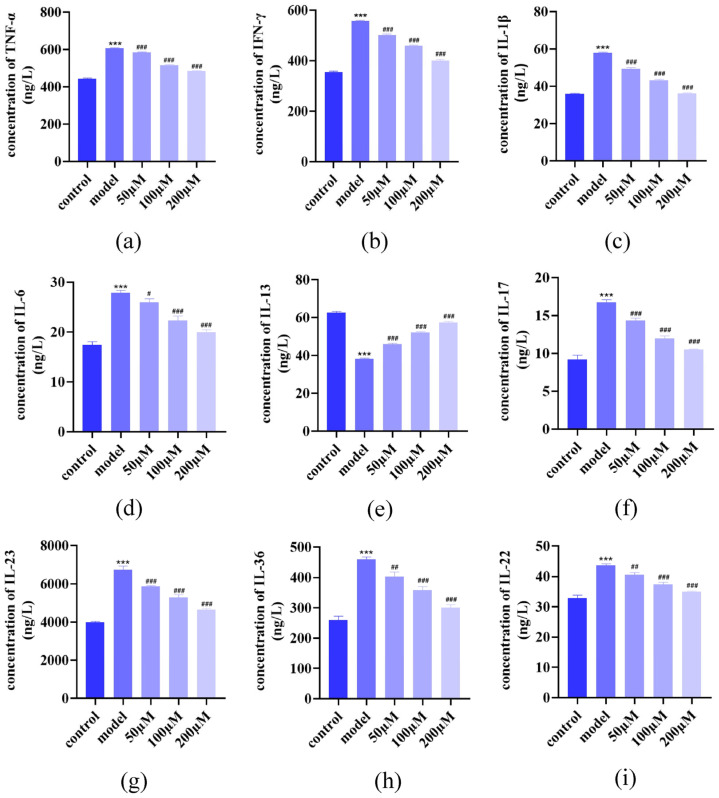
AST modulates cytokine profiles in IL-17-stimulated HaCaT cells. (**a**–**i**) Quantitative analysis of cytokine secretion profiles using ELISA, demonstrating AST-mediated regulation of TNF-α, IFN-γ, IL-1β, IL-6, IL-13, IL-17, IL-23, IL-36, and IL-22 levels in cell culture supernatants. Data are expressed as mean ± SD (*n* = 3). *** *p* < 0.001 versus control group; ^#^ *p* < 0.05, ^##^ *p* < 0.01, ^###^ *p* < 0.001 versus IL-17-induced model group.

Enzyme-linked immunosorbent assay (ELISA) analysis demonstrated an increase in pro-inflammatory cytokines (IL-1β, IL-6, IL-17, IL-23, IL-36, TNF-α, interferon (IFN)-γ, IL-22) and a decrease in anti-inflammatory IL-13. AST dose-dependently suppressed pro-inflammatory cytokine release while enhancing IL-13 secretion (Figure 4a–i), demonstrating its immunomodulatory potential in regulating cytokine networks.

### 2.4. AST Restored GSH, MDA, and C11 Levels in IL-17-Damaged HacaT Cells

**Figure 5:**
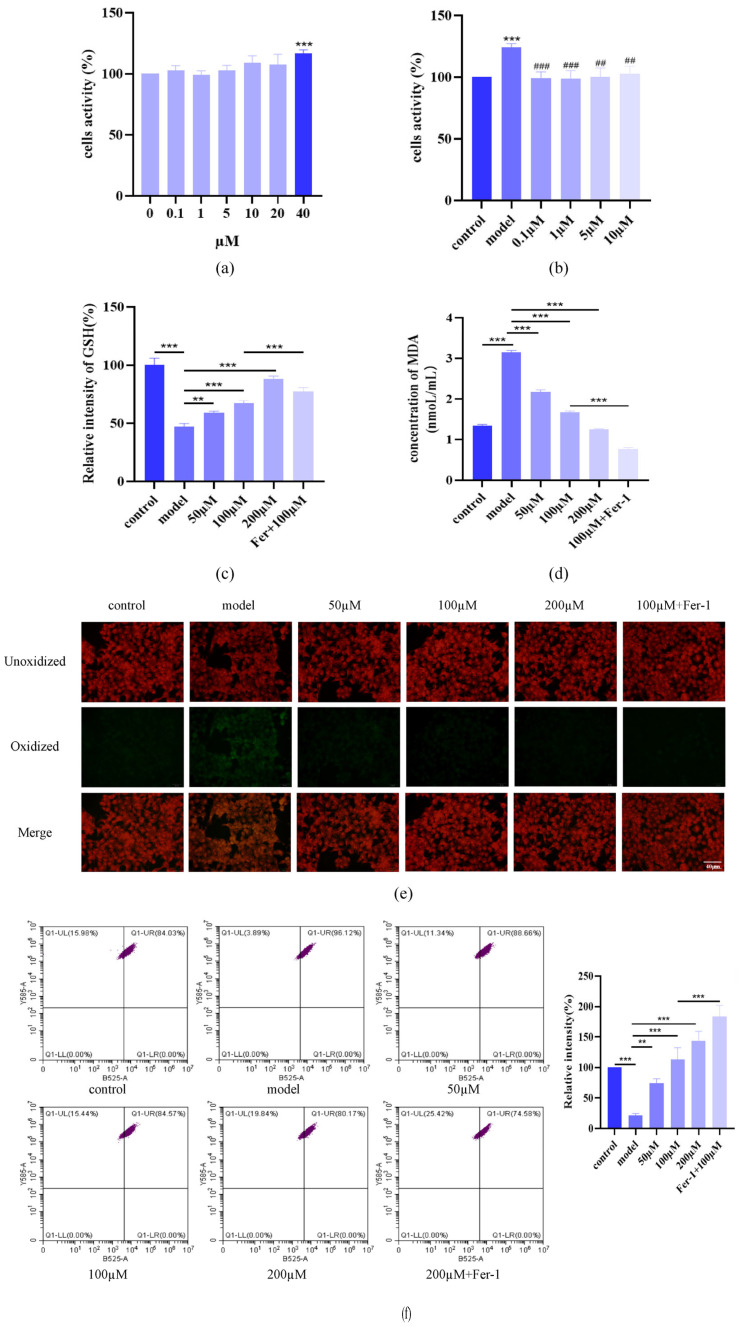
AST restores redox homeostasis in IL-17-stimulated HaCaT keratinocytes. (**a**) Cytotoxicity assessment of Fer-1 in HaCaT cells (*** *p* < 0.001). (**b**) Dose-dependent inhibition of IL-17-induced hyperproliferation by Fer-1 (*** *p* < 0.001 versus control group; ^##^ *p* < 0.01, ^###^ *p* < 0.001 versus IL-17-induced model group). (**c**) Synergistic enhancement of GSH levels by combined AST and Fer-1 treatment (** *p* < 0.01, *** *p* < 0.001). (**d**) Combined treatment-mediated reduction in MDA content (*** *p* < 0.001). (**e**) Fluorescence microscopic visualization of lipid peroxidation using BODIPY 581/591 C11 staining, showing dose-dependent restoration of red/green fluorescence ratio. (**f**) Flow cytometric quantification of lipid peroxides, demonstrating dose-dependent reduction by AST treatment (** *p* < 0.01, *** *p* < 0.001). Data are expressed as mean ± SD (*n* = 3).

To explore AST’s role in ferroptosis, we first assessed the cytotoxicity of ferrostatin-1 (Fer-1), a ferroptosis inhibitor, in HaCaT cells. MTT assays confirmed that Fer-1 concentrations ≤ 20 μM did not affect cell proliferation (Figure 5a). Based on dose–response analysis, 10 μM Fer-1 was selected for subsequent experiments as it effectively suppressed IL-17-induced hyperproliferation without compromising viability (Figure 5b). AST treatment dose-dependently increased intracellular GSH levels (Figure 5c) and reduced MDA content and lipid peroxidation (Figure 5d–f). Notably, the combination of AST and Fer-1 synergistically restored redox homeostasis, suggesting enhanced protection against IL-17-induced oxidative damage of ferroptosis.

### 2.5. AST Restored Fe^2+^ Levels, Mitochondrial Structure, and Protein Levels in IL-17-Damaged HaCaT Cells

**Figure 6:**
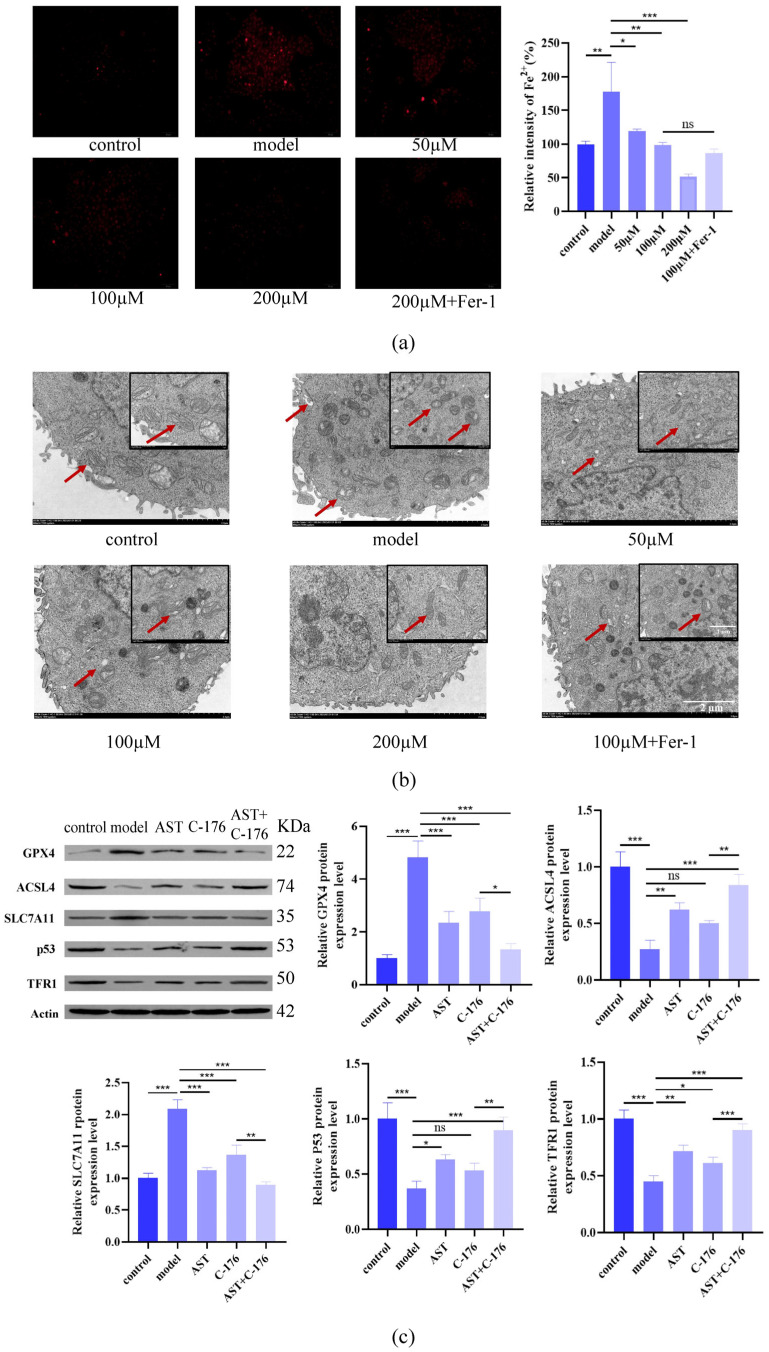
AST restores Fe^2+^ homeostasis, mitochondrial integrity, and cGAS-STING signaling in IL-17-stimulated HaCaT keratinocytes. (**a**) Quantitative analysis of intracellular Fe^2+^ levels using FeRhoNox-1 staining and fluorescence microscopy, demonstrating dose-dependent reduction by AST and synergistic effects with Fer-1 pretreatment (* *p* < 0.05, ** *p* < 0.01, *** *p* < 0.001, “ns” denotes non-significant). (**b**) Transmission electron microscopic visualization of mitochondrial ultrastructure, showing AST-mediated restoration of cristae organization and membrane integrity. The red arrows point to changes in mitochondrial ultrastructure. (**c**) Western blot analysis of cGAS-STING pathway components, including STING, p-STING, cGAS, IRF3, p-IRF3, TBK1, p-TBK1, and IFN-β protein expression levels (* *p* < 0.05, ** *p* < 0.01, *** *p* < 0.001, “ns” denotes non-significant). Data are expressed as mean ± SD (*n* = 3).

Fluorescence microscopy and ImageJ analysis revealed that AST dose-dependently reduced intracellular Fe^2+^ accumulation in IL-17-stimulated HaCaT cells (Figure 6a). However, Fer-1 did not significantly enhance AST’s iron-chelating effects, indicating that AST’s iron regulation is independent of Fer-1.

Transmission electron microscopy (TEM) analysis showed that the cells in the control group exhibited elongated mitochondria with well-organized cristae and intact membranes, whereas IL-17-treated cells displayed rounded mitochondria with disorganized cristae, membrane disruption, and extensive vacuolization. AST treatment dose-dependently restored mitochondrial architecture and reduced cytoplasmic vacuoles (Figure 6b). The combination of AST and Fer-1 provided superior mitochondrial protection, suggesting complementary mechanisms in preserving mitochondrial integrity.

To explore whether ferroptosis in inflammatory cells is initiated via activation of the cGAS-STING signaling pathway, we analyzed the expression of key ferroptosis regulators in HaCaT cells treated with AST and the STING inhibitor C-176. Western blot analysis revealed that AST and C-176 co-treatment significantly downregulated anti-ferroptotic proteins (GPX4, SLC7A11) while upregulating pro-ferroptotic markers (ACSL4, p53, TFR1; Figure 6c). The combination of AST and C-176 exhibited maximal suppression of ferroptosis, indicating that STING inhibition enhanced AST’s anti-ferroptotic effects, likely through modulation of the iron metabolism and lipid peroxidation pathways.

### 2.6. AST Inhibited cGAS-STING Pathway Activation

**Figure 7:**
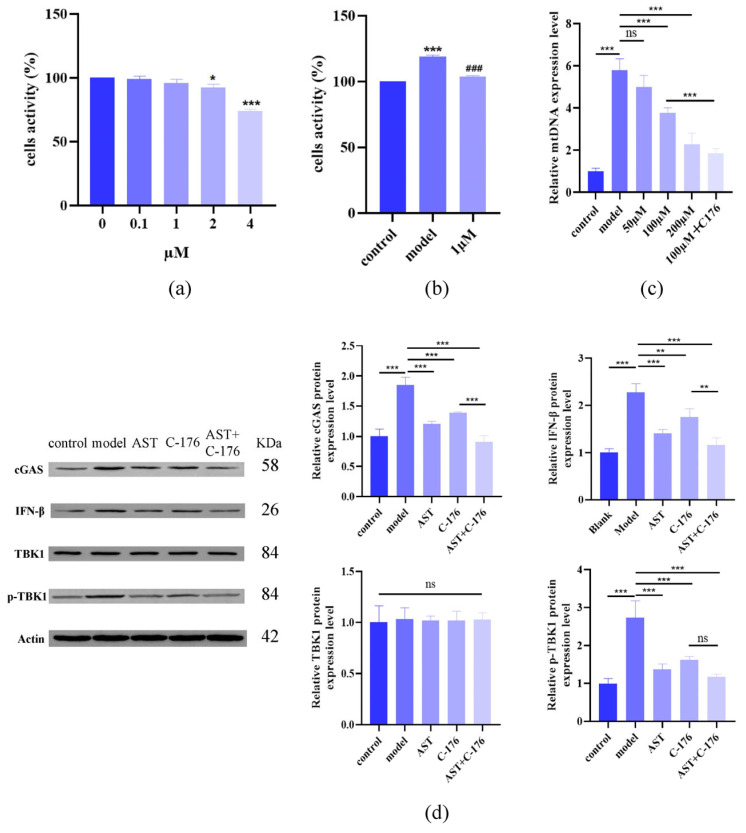

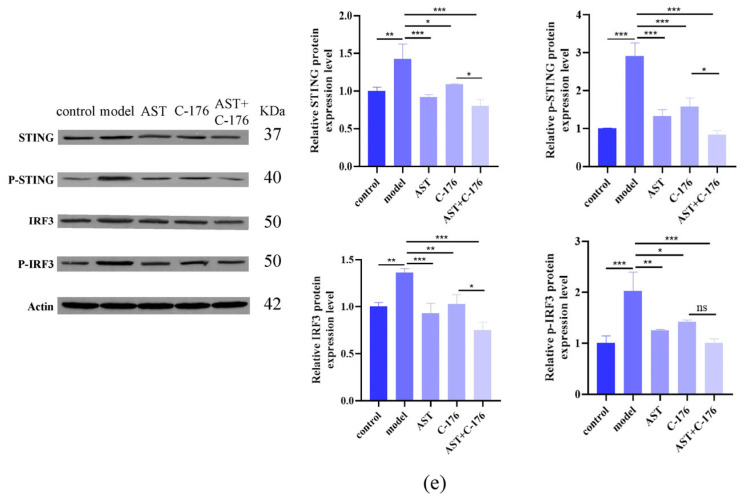
Astilbin (AST) inhibits cGAS-STING pathway activation in IL-17-stimulated HaCaT keratinocytes. (**a**) Cytotoxicity assessment of C-176 (STING inhibitor) in HaCaT cells across concentration gradients (* *p* < 0.05, *** *p* < 0.001 versus control group). (**b**) Suppression of IL-17-induced hyperproliferation by 1 μM C-176 (*** *p* < 0.001 versus control group; ^###^ *p* < 0.001 versus IL-17-induced model group). (**c**) qPCR analysis of mtDNA content, demonstrating synergistic reduction by AST and C-176 combination therapy (*** *p* < 0.001, “ns” denotes non-significant). (**d**,**e**) Western blot analysis of cGAS-STING pathway components, including STING, p-STING, cGAS, IRF3, p-IRF3, TBK1, p-TBK1, and IFN-β protein expression levels, showing dose-dependent inhibition by AST and enhanced suppression with C-176 co-treatment (* *p* < 0.05, ** *p* < 0.01, *** *p* < 0.001, “ns” denotes non-significant). Data are expressed as mean ± SD (*n* = 3).

To investigate the role of cGAS-STING signaling, we employed the STING inhibitor C-176 (1 μM, non-cytotoxic; Figure 7a,b). The results of qPCR analysis revealed the accumulation of cytoplasmic mtDNA in IL-17-treated cells (Figure 7c), confirming mitochondrial-damage-driven cGAS-STING activation. Western blot analysis showed that AST downregulated key pathway components (STING, p-STING, cGAS, IRF3, p-IRF3, IFN-β) without affecting total TBK1 (Figure 7d,e). Notably, combined treatment with AST and C-176 synergistically suppressed the cGAS-STING signaling, suggesting dual-pathway targeting as a therapeutic strategy.

## 3. Discussion

The psoriatic inflammation model in HaCaT cells exhibited characteristic features including cellular and mitochondrial damage, oxidative stress, and immune dysregulation [38,39]. Our findings demonstrated that AST treatment dose-dependently mitigated these pathological alterations, reducing oxidative stress markers, restoring mitochondrial function, and attenuating inflammatory cytokine production. Psoriatic pathogenesis involves complex interactions between innate and adaptive immunity [2,40]. The inflammatory cascade activates dendritic cells to secrete IL-23 and IL-12, which in turn stimulate keratinocytes to produce pro-inflammatory mediators including IL-1β, IL-6, and TNF-α. Concurrently, adaptive immune responses are initiated, with Th1-derived IFN-γ and Th17-derived IL-17/IL-22 playing pivotal roles in disease progression [41,42]. Our cytokine profiling demonstrated that AST significantly decreased the levels of key inflammatory mediators, including IL-1β, IL-6, TNF-α, IFN-γ, and IL-17 in IL-17-stimulated HaCaT cells. These findings align with previously reported results [43,44]. Interestingly, we observed decreased IL-13 secretion in AST-treated cells, contrasting with the elevated IL-13 levels reported in psoriatic lesions in [45]. We hypothesized that the elevated IL-13 levels observed in psoriatic lesions may primarily result from the recruitment of immune cells, which are known to secrete higher amounts of IL-13 [46]. In contrast, the secretion of IL-13 by HaCaT cells might indicate cell-type-specific behavior, potentially exerting an anti-inflammatory role in this context. However, AST treatment significantly attenuated IL-13 levels in HaCaT cells, which may correlate with C-C motif chemokine ligand 26 (CCL26). Histopathological analysis reveals pronounced eosinophil infiltration in psoriatic lesions—a phenomenon mechanistically linked to IL-13-mediated CCL26 upregulation [47]. Mechanistically, CCL26 functions as a key eosinophil chemoattractant, driving the spatial recruitment of eosinophils to the psoriatic epidermis and amplifying local inflammation [48,49]. AST significantly reduces the level of IL-13 and may further reduce the level of CCL26 [50]. The dual role of IL-13 in psoriasis exists in the lesion sites of psoriasis, where it can jointly alleviate the inflammatory response. Furthermore, clinical studies have demonstrated that the trend of cytokine changes at the lesion sites of patients with psoriasis is consistent with our research [42,51,52,53,54,55].

Oxidative stress and lipid peroxidation accumulation in inflammatory cells may serve as the molecular basis for ferroptosis induction. Emerging evidence suggests that ferroptotic cell death is characterized by three hallmark features: excessive lipid peroxidation, dysregulated iron metabolism, and severe mitochondrial damage [56,57,58]. In our IL-17-stimulated HaCaT cell model, we observed characteristic ferroptotic alterations, including mitochondrial ultrastructural damage, elevated lipid peroxidation products, decreased Fe^2+^ levels, and dysregulated expression of key ferroptosis regulators such as GSH. These findings demonstrated that IL-17 induced ferroptosis in keratinocytes, while AST treatment dose-dependently attenuated these ferroptotic changes. Based on these observations, we proposed that AST might exert its anti-inflammatory effects, at least in part, through inhibition of ferroptosis. This protective mechanism likely involves multiple pathways, which lead to a reduction of oxidative stress and lipid peroxidation, restoration of iron homeostasis, and preservation of mitochondrial integrity. The ability of AST to modulate these key aspects of ferroptosis suggests its potential as a therapeutic agent for inflammatory skin disorders characterized by ferroptotic cell death.

Mitochondrial damage in inflammatory cells leads to the release of mtDNA into the cytoplasm, which can be recognized by cGAS and subsequently activates the cGAS-STING signaling pathway, thereby amplifying inflammatory responses through type I interferon production [59]. In our IL-17-stimulated HaCaT cell model, we observed increased cytoplasmic mtDNA levels, suggesting potential activation of the cGAS-STING pathway. Mechanistic studies revealed that AST treatment significantly downregulated key components of the cGAS-STING signaling cascade, including cGAS, STING, and downstream effector IFN-β. These findings demonstrated that AST could effectively inhibit cGAS-STING pathway activation in psoriatic keratinocytes. Based on these observations, we propose that AST exerts its anti-inflammatory effects, through modulation of the cGAS-STING signaling axis. The ability of AST to target multiple nodes within this pathway highlights its potential as a therapeutic agent for inflammatory disorders characterized by cGAS-STING pathway activation.

Emerging evidence suggests a complex interplay between ferroptosis and the cGAS-STING signaling pathway in modulating inflammatory responses. Reactive ROS accumulation during inflammation can trigger ferroptotic cell death, which in turn exacerbates oxidative stress, induces mitochondrial damage, and activates the cGAS-STING pathway through mtDNA release. This creates a self-amplifying cycle that perpetuates inflammatory signaling [25,26,60]. To investigate this potential crosstalk, we employed the STING inhibitor C-176 in combination with AST treatment. Our findings indicated that both C-176 and AST significantly downregulated key ferroptosis regulators, including GPX4, SLC7A11, and TFR1. These results provide experimental evidence supporting the functional connection between ferroptosis and cGAS-STING signaling in inflammatory cells. While our study sheds light on this molecular interplay, several mechanistic questions remain unresolved: the precise molecular mediators linking ferroptosis to cGAS-STING activation, the temporal dynamics of this crosstalk during inflammatory progression, and the cell-type specificity of these interactions. Further investigation will be necessary to fully elucidate the complex relationship between these two pathways and their collective impact on inflammatory diseases.

In summary, our data demonstrated that AST effectively suppressed IL-17-induced hyperproliferation in HaCaT keratinocytes, mitigated cellular and mitochondrial damage, and restored cytokine homeostasis. Mechanistically, AST exerted its anti-inflammatory effects through dual modulation of ferroptosis and the cGAS-STING signaling pathway. These results position AST as a promising therapeutic candidate for psoriasis treatment, with the potential to address multiple pathological features of the disease. However, several critical questions remain to be addressed: (1) the precise molecular targets of AST in regulating ferroptosis and cGAS-STING signaling, (2) the in vivo efficacy and safety profile of AST in psoriatic models, and (3) the potential synergistic effects of AST with existing psoriasis therapies. Future studies incorporating advanced omics approaches, genetic manipulation, and preclinical models will be essential to fully elucidate AST’s therapeutic potential and mechanism of action in psoriasis.

## 4. Materials and Methods

### 4.1. Chemicals and Reagents

Astilbin was purchased from konoscience (Beijing, China). (3-[4, 5-Dimethyl-2-thiazolyl]-2, 5-diphenyltetrazolium bromide (MTT) was purchased from LABLEAD (Beijing, China). Dimethyl sulfoxide (DMSO) was purchased from Sigma-Aldrich (St Louis, MO, USA). The LDH Cytotoxicity Assay Kit, Reactive Oxygen Species Assay Kit, DAPI, Mitochondrial membrane potential assay kit with JC-1, Total Glutathione Assay Kit, and Lipid Peroxidation MDA Assay Kit were obtained from Beyotime (Shanghai, China). The Lipid Peroxidation Assay Kit with BODIPY 581/591 C11 and FeRhoNox-1 (Fe^2+^ indicator) were purchased from Abmole (Houston, TX, USA). Glutaraldehyde Fixative (2.5%, for electron microscopy) was obtained from Biosharp (Shanghai, China). IFN-γ, IL-23, IL-23, IL-6, IL-1β, IL-13, IL-17 (A), IL-22, TNF-α, and IL-36 ELISA kits (Human) were purchased from MAISHA INDUSTRIES (Taizhou, China). The Malondialdehyde Assay Kit, Total Superoxide Dismutase Assay Kit with WST-8 ATP, and Content Assay Kit with Molybdophosphoric were purchased from AIDISHENG (Nanjing China). Phosphate-buffered saline (PBS) was obtained from VivaCell (Shanghai, China). Ferrostatin-1 was obtained from MCE (Monmouth Junction, NJ, USA). Anti-Phospho-STING (Ser366) (D7C3S) antibody, Anti-β-Actin antibody, Anti-NAK/TBK1 antibody, Anti-NAK/TBK1 (phospho S172) antibody, Anti-STING antibody, Anti-IRF3 antibody, Anti-IRF3 (phospho S386) antibody, and Anti-IFN beta Receptor beta/AF-1antibody were purchased from Abcam (San Francisco, CA, USA). IL-17, Anti-STING antibody, Anti-ACSL4/FACL4 antibody, Anti- cGAS antibody, Anti GPX4 antibody, Anti-SLC7A11/xCT antibody, and Anti-TERF1 antibody were obtained from Proteintech (Wuhan, China). The BCA Protein Assay Kit was purchased from UtiBody (Tianjin, China). Tris-glycine-SDS TAE Buffer, Tris-Glycine Transfer Buffer, and SDS-PAGE Gel Preparation Kit were purchased from BOSTER (Wuhan, China). Fast Blocking Western and Primary & Secondary Antibody Diluent for WB were purchased from YEASEN (Shanghai, China). SDS-PAGE loading buffer, 5 × (with DTT), ColorMixed Protein Marker (11-180KD), and Super ECL Plus were purchased from BIORIGIN (Beijing, China). The Mitochondrial extraction kit and Animal Tissues/Cells Genomic DNA Extraction Kit were obtained from Solarbio (Beijing, China). 2 × SYBR Green qPCR Master Mix (None ROX) was purchased from Servicebio (Wuhan, China).

### 4.2. Cell Culture, Treatments, and Inflammation Model Establishment

The HaCaT keratinocyte cell line was obtained from Procell Life Science (Wuhan, China, originated from the normal skin surrounding the lesion of a 62-year-old male with melanoma). The HaCaT cells were cultured in DMEM supplemented with 10% fetal bovine serum (FBS) and antibiotics, which were purchased from FuHeng (Shanghai, China), at 37 °C in a 5% CO_2_ atmosphere. To establish a psoriasis-like inflammation model, HaCaT cells were treated with 120 ng/mL recombinant human IL-17 in complete culture medium for 24 h, following optimized induction protocols [61,62].

### 4.3. MTT Assay for Cell Viability

HaCaT cells were seeded in 96-well plates at a density of 1 × 10^4^ cells/well and divided into five groups: control, IL-17-induced model, and AST treatment (50, 100, 200 µM) groups. After treatment, 20 µL of MTT reagent (5 mg/mL) was added to each well, followed by incubation for 4 h at 37 °C. Subsequently, the supernatant was removed and the formazan crystals dissolved in 100 µL of DMSO. Absorbance was measured at 490 nm using a microplate reader to determine cell viability.

### 4.4. Lactate Dehydrogenase (LDH) Release Assay

HaCaT cells were seeded in 96-well plates at 1 × 10^4^ cells/well and grouped as described above. After treatment, plates were centrifuged at 400× *g* for 5 min, and 120 µL of supernatant was transferred to a new plate. LDH activity was quantified by adding 60 µL of detection reagent to each well, followed by incubation in the dark at 25 °C for 30 min. Absorbance was measured at 490 nm using a microplate spectrophotometer.

### 4.5. Intracellular SOD and ATP Quantification

HaCaT cells were seeded in 6-well plates at 5 × 10^5^ cells/well and grouped as above. The cells were washed with PBS, lysed using a sonicator (200 W, 3 s pulse/10 s interval, 30 cycles), and incubated on ice for 10 min. Lysates were centrifuged at 12,000 rpm for 10 min at 4 °C, and supernatants were stored at −20 °C. ATP content was measured spectrophotometrically at 700 nm, and SOD activity was quantified at 450 nm using commercial kits according to the manufacturer’s protocols.

### 4.6. Detection of Intracellular ROS Levels

HaCaT cells were seeded in 6-well plates at 5 × 10^5^ cells/well and grouped as above. After treatment, cells were washed with PBS, trypsinized, and resuspended in 10 µM DCFH-DA working solution. The cells were then incubated in the dark at 37 °C for 20 min with intermittent agitation. Following three PBS washes, intracellular ROS levels were quantified by flow cytometry based on DCF fluorescence intensity, normalized to untreated controls. For fluorescence microscopy, cells were incubated with 1 mL of DCFH-DA working solution (10 µM) under standard culture conditions for 20 min, washed three times with PBS, and visualized using a fluorescence microscope.

### 4.7. ELISA Detection of Cytokines

HaCaT cells were seeded in 6-well plates at 5 × 10^5^ cells/well and grouped as above. Conditioned media were collected and centrifuged at 3000× *g* for 20 min at 4 °C to remove cellular debris. The supernatants were aliquoted and stored at −20 °C until analysis. Cytokine levels (IL-1β, IL-6, IL-17, IL-23, IL-36, TNF-α, IFN-γ, IL-13, IL-22) were quantified using commercial ELISA kits according to the manufacturer’s protocols. Absorbance was measured using a microplate spectrophotometer, and cytokine concentrations were calculated based on standard curves.

### 4.8. Measurement of Mitochondrial Membrane Potential

HaCaT cells were seeded in 6-well plates at 5 × 10^5^ cells/well and grouped as described above. After treatment, cells were washed with PBS, trypsinized, and resuspended in complete medium at a density of 1 × 10^5^–6 × 10^5^ cells/mL. Cells were stained with 0.5 mL JC-1 working solution and incubated at 37 °C in 5% CO_2_ for 20 min in the dark. After centrifugation (600× *g*, 4 °C, 3 min), cells were washed twice with JC-1 staining buffer and resuspended in 300 µL of buffer for immediate flow cytometric analysis.

### 4.9. Observation of Mitochondrial Morphology

HaCaT cells were seeded in 6-well plates at 5 × 10^5^ cells/well and divided into six groups: control, IL-17-induced model, AST treatment (50, 100, 200 µM), and Fer-1 rescue groups. For ultrastructural analysis, cells were fixed in 2.5% glutaraldehyde (0.1 M phosphate buffer, pH 7.4) at 4 °C for 2 h, followed by post-fixation in 1% osmium tetroxide for 2 h at 4 °C. Samples were dehydrated through an ethanol gradient (50%, 70%, 90%, 100%) and transitioned to acetone. Infiltration was performed with epoxy resin mixtures (acetone: resin = 3:1 for 2 h, 1:1 for 3 h, 1:3 for 3 h), followed by pure resin embedding overnight. Polymerization was achieved by stepwise heating (35 °C → 60 °C → 80 °C, 5 h per step). Ultrathin sections (70–90 nm) were cut using an ultramicrotome, double-stained with uranyl acetate and lead citrate, and imaged using transmission electron microscopy at 80 kV.

### 4.10. Quantification of Intracellular MDA and GSH Levels

HaCaT cells were seeded in 6-well plates at 5 × 10^5^ cells/well and grouped as above. For MDA quantification, cells were lysed in RIPA buffer with protease inhibitors, and lysates were centrifuged at 12,000 rpm for 15 min at 4 °C. Supernatants (100 µL) were mixed with 200 µL MDA detection reagent, incubated in a boiling water bath (95–100 °C) for 15 min, and cooled to room temperature. After centrifugation (1000× *g*, 10 min), absorbance was measured at 532 nm. For GSH quantification, cells were lysed in protein removal reagent S, subjected to freeze–thaw cycles, and centrifuged at 10,000× *g* for 10 min at 4 °C. Supernatants were analyzed at 412 nm using an enzymatic recycling assay.

### 4.11. Detection of Intracellular Lipid Peroxides (C11-BODIPY)

HaCaT cells were seeded in 6-well plates at 5 × 10^5^ cells/well and grouped as above. After treatment, cells were washed with PBS, trypsinized, and resuspended in BODIPY 581/591 C11 working solution (2 µM). The cells were then incubated at 37 °C in 5% CO_2_ for 20 min in the dark, washed twice with PBS, and analyzed by flow cytometry. For fluorescence microscopy, cells were stained with 1 mL BODIPY 581/591 C11 solution, incubated under the same conditions, washed three times with PBS, and imaged.

### 4.12. Detection of Intracellular Fe^2+^ Levels

HaCaT cells were seeded in 6-well plates at 5 × 10^5^ cells/well and grouped as above. After treatment, the cells were washed with PBS and incubated with 1 mL FeRhoNox-1 fluorescent probe (5 µM) at 37 °C in 5% CO_2_ for 30 min in the dark. Cells were washed three times with PBS, and Fe^2+^ levels were visualized using fluorescence microscopy. Fluorescence intensity was quantified using ImageJ software (version 1.54m), with data normalized to untreated controls.

### 4.13. Quantitative PCR (qPCR) Detection of mtDNA

HaCaT cells were seeded in 6-well plates at 5 × 10^5^ cells/well and grouped as above. After treatment, cells were washed with PBS, trypsinized, and centrifuged at 12,000× *g* for 10 min at 4 °C. Mitochondria were isolated using a commercial extraction kit, and genomic DNA was purified. qPCR was performed using 2× SYBR Green Master Mix and the following primers:

mt-F: 5′-CCTCCCATTCATTATCGCCGCCCTTGC-3′

mt-R: 5′-GTCTGGGTCTCCTAGTAGGTCTGGGAA-3′

### 4.14. Western Blot Analysis

HaCaT cells were seeded in 6-well plates at 5 × 10^5^ cells/well and grouped as above. After treatment, cells were lysed in RIPA buffer with protease/phosphatase inhibitors and centrifuged at 12,000× *g* for 30 min at 4 °C. The protein expression levels of ferroptosis regulators (GPX4, ACSL4, SLC7A11, p53, TFR1) and cGAS-STING pathway components (STING, p-STING, cGAS, TBK1, p-TBK1, IRF3, p-IRF3, IFN-β) were analyzed by Western blot using β-actin as a loading control. Densitometric analysis was performed using Image Lab software.

### 4.15. Data Analysis

All experiments were performed in triplicate with three independent biological replicates. Data are presented as mean ± SD and analyzed using GraphPad Prism (v8.0). Statistical comparisons were performed using one-way ANOVA followed by Tukey’s post hoc test. A *p*-value < 0.05 was considered statistically significant.

## 5. Conclusions

This study investigated the novel therapeutic mechanism of AST in IL-17-stimulated HaCaT keratinocytes, focusing on its dual regulation of ferroptosis and cGAS-STING signaling. AST ameliorates ferroptosis progression by suppressing IL-17-induced cGAS-STING pathway activation in HaCaT keratinocytes, which consequently inhibits psoriatic manifestations through attenuating cytokine dysregulation and mitigating oxidative stress. By systematically exploring these pathways, we provide the first evidence of AST’s multimodal action against psoriatic inflammation, offering new insights into its therapeutic potential.

## Data Availability

The original contributions presented in the study are included in the article; further inquiries can be directed to the corresponding authors.

## Abbreviations

The following abbreviations are used in this manuscript:

AST	Astilbin
cGAS-STING	GMP-AMP synthase-stimulator of interferon genes
MDA	malondialdehyde
GSH	glutathione
mtDNA	mitochondrial DNA
DAMPs	damage-associated molecular patterns
DMSO	Dimethyl sulfoxide
FBS	fetal bovine serum
LDH	Lactate Dehydrogenase
qPCR	quantitative PCR
TBK1	TANK-binding kinase 1
IRF3	interferon regulatory factor 3
Th17	T helper 17 cell
IL-23	interleukin-23
IL-17	interleukin-17
ROS	reactive oxygen species
TNF-α	tumor necrosis factor-α
MTT	Methylthiazolyldiphenyl-tetrazolium bromide
SOD	Superoxide dismutase
ATP	Adenosine triphosphate
ELISA	Enzyme-linked immunosorbent assay
IFN	interferon
Fer-1	ferrostatin-1
TEM	transmission electron microscopy
CCL26	C-C motif chemokine ligand 26
